# Aichi virus 3C protease modulates LC3- and SQSTM1/p62-involved antiviral response

**DOI:** 10.7150/thno.47077

**Published:** 2020-07-14

**Authors:** Ming-Hsiang Kung, You-Sheng Lin, Tsung-Hsien Chang

**Affiliations:** 1 Department of Medical Education and Research, Kaohsiung Veterans General Hospital, Kaohsiung 81362, Taiwan.; 2 Department and Graduate Institute of Microbiology and Immunology, National Defense Medical Center, Taipei 11490, Taiwan.

**Keywords:** Autophagy flux, LC3, p62, picornavirus, antiviral inflammation

## Abstract

**Rationale:** Autophagy is an essential, homeostatic process by which cells break down their own components, it also contributes to restricting bacterial infection in host defense systems; yet, how autophagy regulates viral infection remains inconclusive. Aichi virus (AiV), belonging to the genus Kobuvirus in the *Picornaviridae* family, causes acute gastroenteritis in human. The role of autophagy-mediated anti-viral activity on AiV infection was investigated in this study.

**Methods:** The effect of autophagy-associated molecules in retinoic acid-inducible gene-I (RIG-I)-like receptor (RLR) antiviral signal axis was analyzed in AiV infected cells by using biochemistry and pharmacologic approaches. In addition, the AiV viral protein regulating autophagy-associated RLR activity was also evaluated.

**Results**: In AiV-infected cells, autophagic flux including the formation of autophagic vacuoles, as well as degradation of microtubule-associated protein light chain 3 (LC3) and sequestosome-1 (SQSTM1/p62) were observed. Ectopic overexpression of LC3 and p62, but not Atg proteins, contributed to RLR antiviral signal axis, shRNA knockdown of LC3 and p62 led to a downregulation of antiviral inflammation. Moreover, AiV infection inhibited double-stranded RNA (dsRNA)-activated RLR activity by the viral protein 3C protease but not H42D, C143S protease dead mutants. AiV 3C protease caused the degradation of LC3 and p62, and also RLR signal proteins.

**Conclusion:** This study reveals a possible mechanism of autophagy-associated proteins regulating virus replication. Maintaining a cellular level of LC3 and p62 during the viral infection period might help restrict virus replication. Although, AiV 3C protease dampens the LC3 and p62-mediated host antiviral machinery for AiV replication. Results obtained provide a better understanding of the molecular pathogenesis of AiV for developing methods of prevention and treatment.

## Introduction

Aichi virus (AiV), belonging to the *Picornaviridae* family, is a small, round-structured, non-enveloped virus with a single-stranded and positive-sense RNA genome 1, 2*.* The AiV genome organization -- 5' UTR-leader protein-3 structural proteins (viral protein 0 [VP0], VP3 and VP1)-7 nonstructural proteins (2A, 2B, 2C, 3A, 3B, 3C and 3D)-3' UTR -- is identical to that of Kobuvirus 3. AiV infection results mainly in acute gastroenteritis and extra intestinal manifestations such as purulent conjunctivitis or respiratory symptoms in human; however, subclinical infection may be more common than clinically manifested disease 4. Seroepidemiologic studies in different countries showed a high AiV antibody prevalence, which also implicates differential general circulation and infection of the virus in different human populations 4. AiV has been detected in various types of environmental samples, such as sewage, river water, groundwater, and shellfish, suggesting potential transmission of AiV by fecal-oral routes through contaminated food or water 5.

Secretion of type I interferons (IFNs) and inflammatory cytokines can be triggered by nuclear acid of virus replication products, and toll-like receptor (TLR) ligands 6. These cytokines not only inhibit virus replication in infected cells but also regulate induction of adaptive immunity, leading to swift eradication of viruses. Viral double-stranded (dsRNA) can be detected by a group of host cellular sensor proteins defined as retinoic acid inducible gene-I (RIG-I)-like receptors (RLRs) including RIG-I, melanoma differentiation-associated protein 5 (MDA5) and laboratory of genetics and physiology gene 2 (LGP2). One of the mechanisms is that RIG-I or MDA5 binds with dsRNA and then transfers signals to the mitochondria antiviral signaling protein (MAVS) for activation of IFN regulatory factor 3 (IRF3), IRF7 or NFκB, leading to type I IFN expression 6. Virologic analysis revealed that various cell types were susceptible to AiV infection, and the IFN against the AiV was also demonstrated 7; however, the pathogenicity mechanism of AiV remains unclear.

The intrinsic cell-physical activities include organelle trafficking, endoplasmic reticulum and mitochondria activities, and autophagy processes 8-12. Autophagy is an essential, homeostatic process by which cells break down their own components. The autophagy pathway proceeds through several phases, namely initiation or isolation membrane formation, vesicle elongation, autophagosome maturation and autophagosome lysosome fusion, eventually resulting in content degradation. Each phase of the autophagic pathway is regulated by multiple factors. Regulatory factors behind initiation include Beclin 1 class III PI3K complex (Beclin 1-VP34-ATG14L-p150) and mammalian target of rapamycin (mTOR) substrate complex (ULK1-ATG13-ATG101-FIP200). The ATG5-ATG12 conjugation system (ATG3-ATG10, ATG5-ATG12-ATH16L1) and LC3-ATG8 conjugation system (ATG4B-LC3-I, ATG3-ATG7-LC3-II) control the phases of elongation and autophagosome maturation. The sequestosome 1/p62-associated ubiquitin system is the major factor influencing final degradation 13.

Autophagy is involved in regulating the host defense system against bacterial and viral infection 14-17. Autophagy facilitates viral sensing by delivering vesicular stomatitis virus (VSV) viral replication intermediates, single-stranded RNA, to lysosomes to trigger endosomal TLR7 activity, leading to type I IFN production in plasmacytoid dendritic cells (pDCs, major type I IFN producers); thus, the Atg5-deficient pDCs and Beclin 1-deficient DCs are unable to produce type I IFN in response to VSV and respiratory syncytial virus (RSV) infection, respectively 18. Ectopic Beclin 1 suppressed Sindbis virus replication in the brain and reduced mouse mortality 19. In macrophages, the autophagy pathway regulates TLR signaling by the cross-talk of Beclin 1 and MyD88 20. The DNA-containing immune complex triggers TLR9-mediated type I IFN production in pDCs through LC3-associated phagocytosis trafficking but not conventional autophagic preinitiation complex, thus revealing a new role for noncanonical autophagy in inflammation 21.

However, autophagy could negatively regulate type I IFN production in non-pDCs. Atg5-Atg12 conjugate directly associated with caspase recruitment domain (CARD) of RIG-I and MAVS, could block retinoic-like-receptor (RLR) signaling and suppress type I IFN production. Consequently, mouse embryonic fibroblasts (MEF) lacking Atg5-Atg12 conjugates over-produced type I IFN in response to VSV infection 22. A similar result was shown in hepatitis C virus infection; the virus replication was suppressed in Atg5-knockdown cells, and in cells treated with the autophagy inhibitors chloroquine (CQ) or bafilomycin A1 (BAF-A1), and treated cells with innate immunity already activated 23, 24. Taken together, these results suggest an inconclusive and sophisticated regulation machinery between autophagy and host defense system.

In view of the importance of autophagy in cellular activity, this study investigates the role of LC3 and p62 in autophagy of AiV-infected cells to understand the pathogenic mechanism involved.

## Methods

### Aichi virus and cells

The human AiV, genome accession no. JX56424 25, was isolated from a newborn with diarrhea in Taiwan and propagated in Vero cells (ATCC: CCL-81, Manassas, VA, USA). Human lung carcinoma A549 cells (ATCC: CCL-185) and HEK293T cells (ATCC: CRL-3216) were cultured in Dulbecco's Modified Eagle Medium (DMEM) with 10% fetal bovine serum (FBS) and 100 μg/ml penicillin/streptomycin (Gibco, ThermoFisher Scientific, Waltham, MA, USA).

### Reagents and antibodies

The reagents used in this study were rapamycin and hydroxychloroquine (HCQ) (Sigma-Aldrich, St. Louis, MO, USA); 3-Methyladenine (3-MA), Torine 1 and bafilomycin A1 (BAF-A1) (Merck Millipore, Burlington, MA, USA). The antibodies used were anti-RIG-I, anti-MAVS, anti-phospho- TANK-binding kinase 1 (anti-phospho-TBK1), anti-TBK1, anti-phospho-IFN regulatory factor 3 (anti-phospho-IRF3), anti-phospho-c-Jun (Ser73), anti-c-Jun, anti-phospho-NFκB p65 and anti-IκBα (Cell Signaling, Danvers, MA), anti-Tumor necrosis factor receptor-associated factor 6 (TRAF6), anti-IRF3, anti-IRF7, and anti-NFκB p65 (Santa Cruz Biotechnology, Santa Cruz, CA), anti-LC3B and anti-p62 (MBL, Nagoya, Japan), anti-FLAG tag, anti-V5 tag, anti-c-Myc tag, anti-GFP, anti-β-Actin and anti-Gapdh (ThermoFisher scientific). The anti-AiV VP1 antibody was described in our previous study 7. dsRNA (polyI:C) was purchased from InvivoGen (San Diego, CA, USA). Turbofect transfection reagent (ThermoFisher Scientific) was used for transfection in cells.

### AiV infection and plaque assay

Cells were replaced with serum-free medium, followed by the addition of AiV at multiplicity of infection (MOI) 5. After 2 h of adsorption at 37 °C, the cell medium was replaced with culture medium. The infected cells were then incubated. To determine virus titers, culture medium from AiV-infected cells was harvested for plaque-forming assays. Various virus dilutions were added to 80% confluent Vero cells and incubated at 37 °C for 2 h. After adsorption, cells were washed and overlaid with 1% agarose containing Eagle's minimum essential medium with 2% fetal bovine serum. After 7-day incubation, cells were fixed with 10% formaldehyde and stained with 0.5% crystal violet.

### Cloning and plasmid preparation

The constructs of AiV viral proteins and their mutants were constructed by using typical molecular cloning and site-directed mutagenesis approaches with specific primers (Table S1). The cellular signaling protein expression vectors, pEF-BOS-RIG-I, pcDNA3.1 V5-MAVS, pcDNA3.1 FLAG-TBK1, pcDNA3.1 FLAG-IKKε, pCMV7.1 3xFLAG-TRAF6, pcDNA3.1 FLAG-IRF3, pcDNA3.1 3XFLAG-IRF7, pcDNA3.1 V5-TRIM12c, pcDNA3.1 HA-MyD88, pcDNA3.1 V5-p62, and pGFP-p62 were also generated or obtained from Addgene (Watertown, MA, USA). pGFP-LC3 expression vector was kindly provided by Dr. Chih-Wen Shu (I-Shou University, Kaohsiung, Taiwan). The endotoxin-free plasmid kit (Qiagen, Venlo, The Netherlands) was employed to prepare the plasmids. The constructs were validated by nucleotide sequencing, and their protein expression in cells was examined using transfection and immunoblotting. The plasmids pMSCV (Murine Stem Cell Virus)-retrovirus (Clontech. Takara, Mountain View, CA, USA) - AiV 3C protease wild-type (WT) and 3C protease dead mutants (H42D and C143S) were constructed, produced in HEK293T cells. The A549 with 3C and mutant-stable expression were transduced by MSCV retrovirus-3C and mutant infection and puromycin (1 μg/ml) selection.

### Short hairpin RNA (shRNA) lentivirus infection

Endogenous LC3 and p62 protein levels were downregulated by shRNAs constructed in the pLKO.1 lentiviral vector (National RNAi Core Facility, Academia Sinica, Taipei, Taiwan). The targeted sequence of LC3 was CGCTTACAGCTCAATGCTAAT and that of p62 was CCTCTGGGCATTGAAGTTGAT. For lentivirus infection, A549 cells (1×10^6^) were infected with lentivirus-containing medium in the presence of polybrene. The infected cells were selected with puromycin (1 μg/ml) for 4 days.

### Immunofluorescence Assay

Mock or AiV-infected (1×10^5^) or polyI: C-transfected cells were fixed with 4% paraformaldehyde for 30 min, then permeabilized with 0.5% Triton X-100 in phosphate buffered saline (PBS) for 10 min, washed with PBS, and blocked with 10% skim milk in PBS for 30 min. The AiV capsid protein VP1 was detected with an anti-AiV VP1 antibody (1:500) 7, followed by Alexa 568-conjugated anti-rabbit IgG antibody (1:1000; ThermoFisher Scientific) each incubated for 1 h at 25 °C. DsRNA was detected by anti-dsRNA antibody, clone rJ2 (Sigma-Aldrich, St. Louis, MO, USA; Cat. No. MABE1134). Cell nuclei were stained with 300 nM 4',6'-diamidino-2-phenylindole (DAPI) for 10 min. The fluorescence signals were observed and captured under a fluorescence microscope (objective 100×; Axio Observer A1, Zeiss, Oberkochen, Germany).

### Quantitative RT-PCR (qRT-PCR)

Total RNA was extracted from cells (1×10^6^) by adding 500 μL Trizol reagent (Invitrogen, Thermo Fisher Scientific); the RNA pellet was resuspended in 30 μL RNase-free distilled water and stored at -80 °C. For the cDNA synthesis, 5 μg of total RNA was used for reverse transcription by using the SuperScript III reverse transcriptase kit (#18080093, Invitrogen, ThermoFisher Scientific) according to the manufacturer's instructions. Real-time PCR was performed using 3 μL cDNA, 3 uM specific primers targeting the genes of interest and 1× (final concentration) SYBR green PCR Master mix (Applied Biosystems, Foster City, CA, USA) in a final reaction volume of 10 μL. Amplification involved using the Applied Biosystems StepOnePlus real-time PCR system involved activation at 95 °C for 20 min, followed by 40 amplification cycles of 95 °C for 3 s and 60 °C for 1 s. Real-time data were analyzed using StepOnePlus software from Applied Biosystems. mRNA expression (fold induction) was quantified by calculating the 2^-ΔΔCt^ value, with reference to level of glyceraldehyde-3-phosphate dehydrogenase (Gapdh), a commonly used housekeeping gene. All qRT-PCR primers were designed by using Primer 3.0 (Applied Biosystems), and the primer sequences are shown in Table S2.

### Western Blot Analysis

Cells (5×10^5^ or 1×10^6^) were lysed in protein lysis buffer (2% SDS, 50 mM Tris-HCl, pH 7.5) containing protease inhibitor and phosphatase inhibitor cocktail (Roche, Basel, Switzerland) 26. Protein concentration was determined by using a Bradford assay kit (BioRad, Hercules, CA, USA). An amount of 50-80 µg protein lysates were separated on 10% SDS-PAGE gel and transferred to polyvinylidene difluoride membranes, which were blocked with 5% milk in Tris-buffered saline (TBS) for 1 h at room temperature, and then incubated with primary antibody overnight at 4 °C. After washing with TBST buffer (TBS with 0.05% Tween X100), the membranes were incubated with horseradish peroxidase-conjugated secondary antibody (Jackson ImmunoResearch Laboratory, West Grove, PA, USA) for 2 h at room temperature and then revealed using enhanced chemiluminescent (ECL) reagent (Advansta, San Jose, CA, USA). Image and emission signal density measurements were quantified by using a BioSpectrum Image System (UVP, Upland, CA).

### Transmission electron microscopy

A549 cells in 10 cm dishes were mock or AiV infected for 24 h. Cells were briefly trypsinized, pelleted, rinsed and resuspended in 2.5% glutaraldehyde fixation buffer (Ems-Chemie, Switzerland). Cell pellets were postfixed in osmium tetroxide, and dehydrated in an ethanol series (50, 70, 80, 90 and 100%) for 15 min at each step. Pellets were then infiltrated and embedded in Spurr's resin (EMS) and polymerized at 72 °C for 12 h. Ultrathin sections (70 nm) were cut by using a Leica EM UC7 system (Wetzlar, Germany) and placed on copper grids. Cells were examined by using a HT7700 transmission Electron Microscope (Hitachi, Tokyo) at 100 kV. Autophagic structures were quantified from images encompassing approximately 4 μm × 5 μm of cell area each. Electron microscopy was performed by Material Analysis Technology Inc. (MA-tek, Hsinchu, Taiwan).

### Luciferase reporter assay

Cells (1 or 2×10^5^) cultured in 12-well plates were transfected with IFN-sensitive response element (ISRE), NFκB, AP-1 or IFNβ-Luc reporter plasmids (400 or 600 ng each). pRL-TK (40 or 60 ng, Promega), encoding *Renilla* luciferase under an HSV thymidine kinase promoter, was used as an internal control. In certain experiments, the viral protein or signal protein expression plasmids were cotransfected into cells. Then, cell lysates were harvested for dual-luciferase assay (Promega). Firefly luciferase activity was normalized to that of *Renilla*.

### Statistical analysis

Data were presented as mean±SD obtained from at least three independent experiments. Significant differences or correction between groups were analyzed by two-tailed Student *t*-test. P < 0.05 was considered statistically significance.

## Results

### AiV induces modest antiviral inflammation response

**Figure 1:**
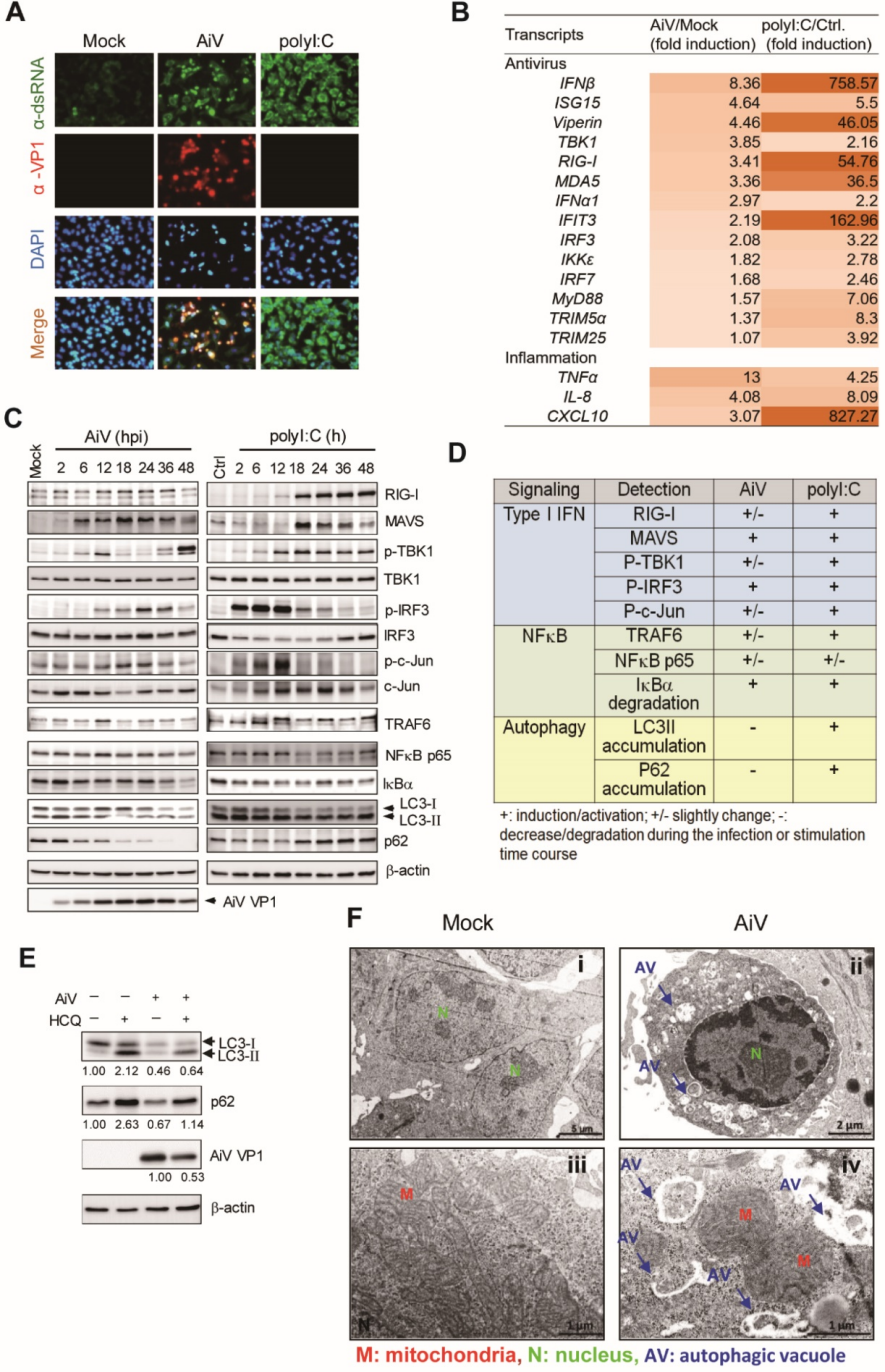
** Assessment of AiV-induced innate immune response and autophagy. (A)** A549 cells were mock infected or infected with AiV (MOI 5) or stimulated by polyI:C for 48 h. The dsRNA and AiV VP1 were detected by immunofluorescence assay with the antibody against anti-dsRNA (green) and anti AiV VP1 (red) **(B)** RT-PCR analysis of the antiviral and inflammation gene induction in A549 cells with mock or AiV infection for 24 h. **(C)** Immunoblotting of RLR pathway and autophagic molecules LC3 and p62 in A549 cells (5×10^5^) stimulated with polyI:C (5 μg) or infected with AiV (MOI 5) for 2 to 48 has indicated. β-actin was the loading control. **(D)** The protein expression of AiV- or polyI:C-challenged cells at 12 or 48 h shown in (C) by comparison to mock or untreated control cells. +, increase; +/-, modest increase; -, no change. **(E)** A549 cells were treated with hydroxychloroquine (HCQ, 10 μM) after AiV adsorption, then incubated for 24 h. Cell lysates were harvested immunoblotting analysis of LC3, p62 and AiV VP1. β-actin was shown as the loading control. **(F)** Ultrastructure of mock (i and iii) and AiV-infected A549 cells (ii and iv) for 24 h observed by transmission electron microscopy. AV, autophagic vacuole; M, mitochondria; N, nucleus. Scale bars: 1 (iii and iv), 2 (ii) or 5 (i) µm.

Host antiviral activation would be triggered by the interaction of dsRNA with TLR3 or RIG-I-like receptors (RLRs) 27. As well as gastroenteritis, AiV also causes lower respiratory tract disease in humans. A549 cells (lung carcinoma) have known to be one of the susceptible cell types to AiV infection 7. To understand whether AiV infection produces dsRNA to activate host antiviral response in A549 cells, we conducted immunofluorescence assay at 24 h post-infection. Along with the VP1 protein expression, dsRNA was also detected in AiV-infected cells. PolyI:C transfection was the dsRNA staining control (Figure 1A). The activation of AiV-mediated antiviral machinery was evaluated by RT-qPCR detection of antiviral inflammation transcripts. As compared with polyI:C, AiV induced a lower level of antiviral gene expression, with huge differences in the level of IFNβ, Viperin, RIG-I, MDA5, IFN induced protein with tetratricopeptide repeats 3 (IFIT3) and CXCL10 between AiV infection and polyI:C stimulation (Figure 1B). Although polyI:C has been known to be a strong inducer of innate immune response, AiV-infected cells showing a low level of antiviral response was unexpected.

To understand whether different levels of antiviral inflammation gene expression was due to different activation level of antiviral signaling triggered by AiV and polyI:C, cell lysates from a time course of AiV infection or polyI:C stimulation were subjected to immunoblotting (Figure 1C), and the changes in signal protein level during the infection/stimulation course is summarized (Figure 1D). Cells with polyI:C stimulation showed a higher level or prolonged induction of RIG-I, phospho-TBK1, phospho-IRF3, phospho-cJun and TRAF6 as compared with those infected by AiV, revealing an inefficient antiviral signaling response that allows AiV replication in cells, shown as AiV VP1 expression (Figure 1C).

The immunoblotting of AiV infection showed an increased level of LC-3II at 2 h post-infection, and degradation of LC3 and p62/SQSTM1 at late times (18-48 h) of infection (Figure 1C-D). The AiV-mediated LC3 and p62 degradation could be blocked by the autophagosome-lysosome fusion inhibitor HCQ (Figure 1E). These results suggest that AiV infection triggered the autophagy process of protein degradation. In addition, TEM images showed numerous phagophores and autophagic vacuoles in AiV-infected cells. The limiting membrane is partially visible as two bilayers separated by a cleft, as detailed in the close-up view, but vacuoles were rarely found with mock infection (Figure 1F). Therefore, that AiV infection triggers autophagy flux and autophagosome formation in cells.

A different pattern of LC3 and p62 immunoblots was observed in polyI:C-stimulated cells, in with LC3-II not degraded and p62 accumulated during the culture period (Figure 1C). Accumulation of LC3 and p62 was found along with the polyI:C-induced strong RLR signaling, suggesting the involvement of LC3 and p62 in the antiviral inflammation pathway.

### Autophagosome inhibitor suppresses AiV replication

**Figure 2:**
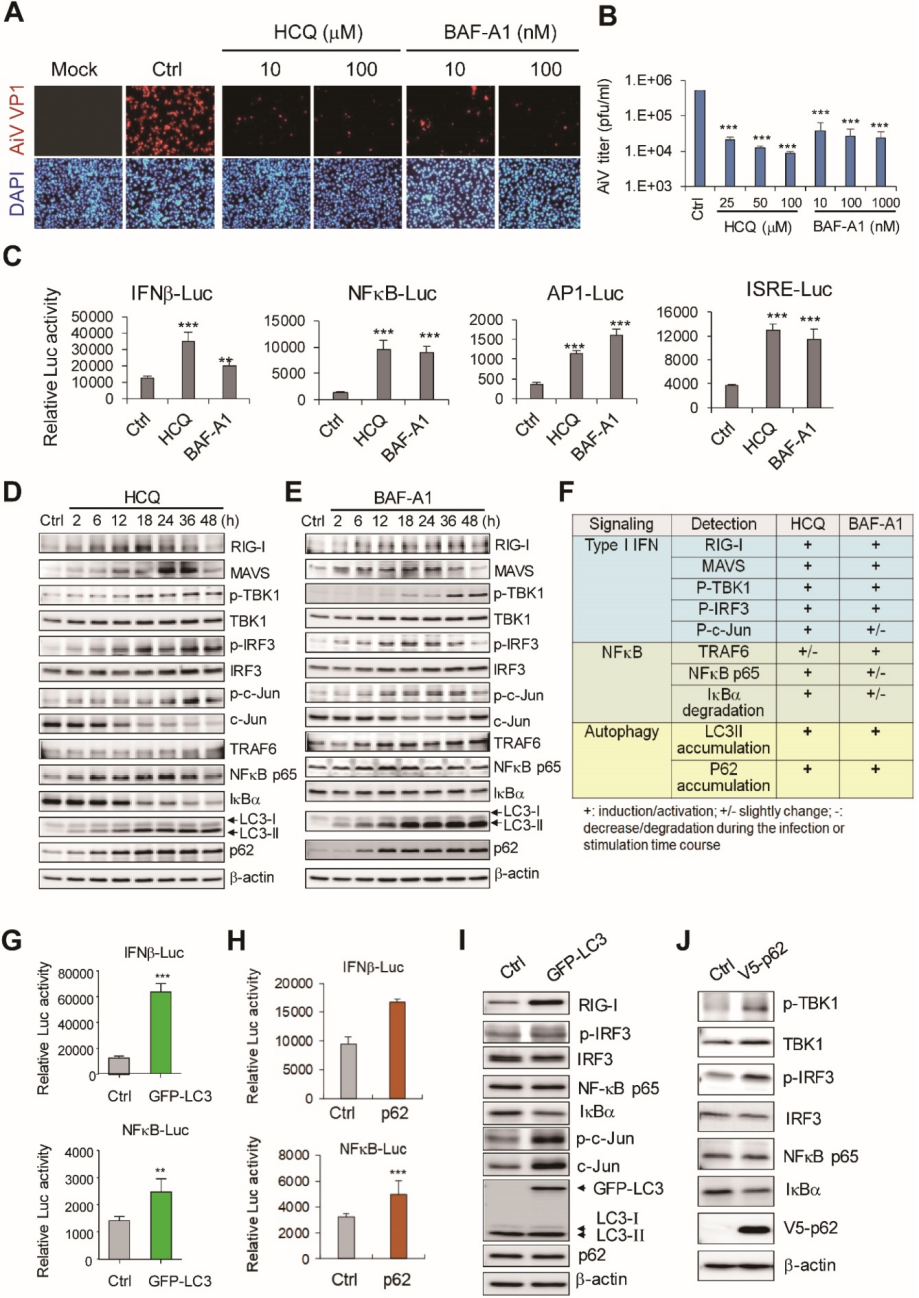
** LC3 and P62 contribute to retinoic acid-inducible gene-I (RIG-I)-like receptor (RLR) activity. (A)** A549 cells were treated with HCQ and BAF-A1 for 24 h before AiV (MOI 5) infection. After 24 h infection, AiV-infected cells were detected by immunofluorescence assay with anti-VP1 antibody (green). DAPI staining (blue) showed the nuclear location. **(B)** Plaque assay of medium harvested from the AiV-infected A549 cells with HCQ or BAF-A1 pretreatment, the calculated virus titers are shown. **(C)** Luciferase reporter assay of IFNβ, NFκB, AP-1 and ISRE in A549 cells (1×10^5^). Cells were treated with HCQ (50 μM) and BAF-A1 (100 nM) for 24 h followed by dual luciferase assay. **(D-E)** Immunoblotting assay of RLR signaling and LC3, p62 proteins in A549 cells, which were treated with HCQ (100 nM) and BAF-A1 (100 nM). **(F)** Protein level changes at 24 h from panels d-e. The data were evaluated by comparison with mock or untreated control cells. +, increase; +/-, modest increase; -, no change. **(G-H)** LC3 and p62 overexpression induced IFNβ and NFκB luciferase reporter activities. **(I-J)** Cell lysates harvested from A549 cells with LC3 and p62 overexpression were immunoblotted with the antibodies for the indicated proteins. Data are presented as mean±SD from three independent tests; **, P < 0.01, ***, P < 0.005 vs untreated group.

In view of different levels of antiviral response and distinct patterns of LC3 and p62 degradation with AiV and ployI:C, the role of LC3 and p62 in regulating cellular antiviral inflammation was investigated. Autophagy inhibitors are widely used in the analysis of biological function of autophagy and clinical trial of cancer therapy 28, 29. Autophagosome-lysosome fusion inhibitors such as HCQ and BAF-A1 block the late-stage autophagy-associated protein degradation, resulting in accumulation of LC3 and p62 in cells. Immunofluorescence assay revealed inhibited AiV infection in HCQ- and BAF-A1-treated cells (Figure 2A). Plaque titration assay also showed that AiV virion production was dose-dependently downregulated by HCQ and BAF-A1 (Figure 2B).

The effect of autophagy activating host antiviral innate immunity was revealed 30, 31. Also, the autophagosome inhibitor HCQ-induced antiviral inflammation against DENV infection 32. Here, we demonstrated that HCQ- and BAF-A1-activated IFNβ, NFκB, AP-1 and ISRE luciferase reporter activity (Figure 2C). In addition, HCQ and BAF-A1 could block LC3 and p62 degradation and activate RLR antiviral activity; RIG-I, MAVS protein levels were increased, phosphorylation of TBK-1 and IRF3 was observed (Figure 2D-F). In view of similar LC3 and p62 protein accumulation in polyI:C-stimulated and HCQ- and BAF-A1-treated cells (Figure 1D and 2F), the role of LC3 and p62 in RLR-mediated antiviral activity was investigated.

### LC3 and p62 overexpression activates RLR signaling axis

Autophagy can regulate TLR signaling 30 through type I IFN production promoted by the interaction of IKKα with LC3 33, and enhancement of nucleotide-binding oligomerization domain-containing protein 2 (NOD-2)-mediated signaling and cytokine production by p62 34. Thus, LC3 and p62 might serve as regulators bridging autophagy and innate immunity. Indeed, reporter assay revealed IFNβ and NFκB luciferase signaling activated by LC3 and p62 overexpression (Figure 2G-H). Immunoblotting revealed that LC3 overexpression also activated the RLR pathway, with increase in phospho-IRF3, c-Jun and RIG-I expression levels, and degradation of IκBα (Figure 2I). Similarly, increased phospho-TBK1, phospho-IRF3 and decreased IκBα were detected in cells with p62 ectopically expression (Figure 2J). Reporter assay on the function of Atg proteins in innate immune signaling showed that Atg proteins were ineffective in activating IFNβ, NFκB, AP-1 and ISRE reporters (Figure S1A). Moreover, unlike p62 and MAVS transfection controls, Atg5 and Atg16L1 overexpression were not able to activate TBK1 and IRF3 (Figure S1B). These data suggested that LC3 and p62 accumulation could be the critical event activating the antiviral machinery in cells.

### LC3 and p62 contribute to the antiviral response

**Figure 3:**
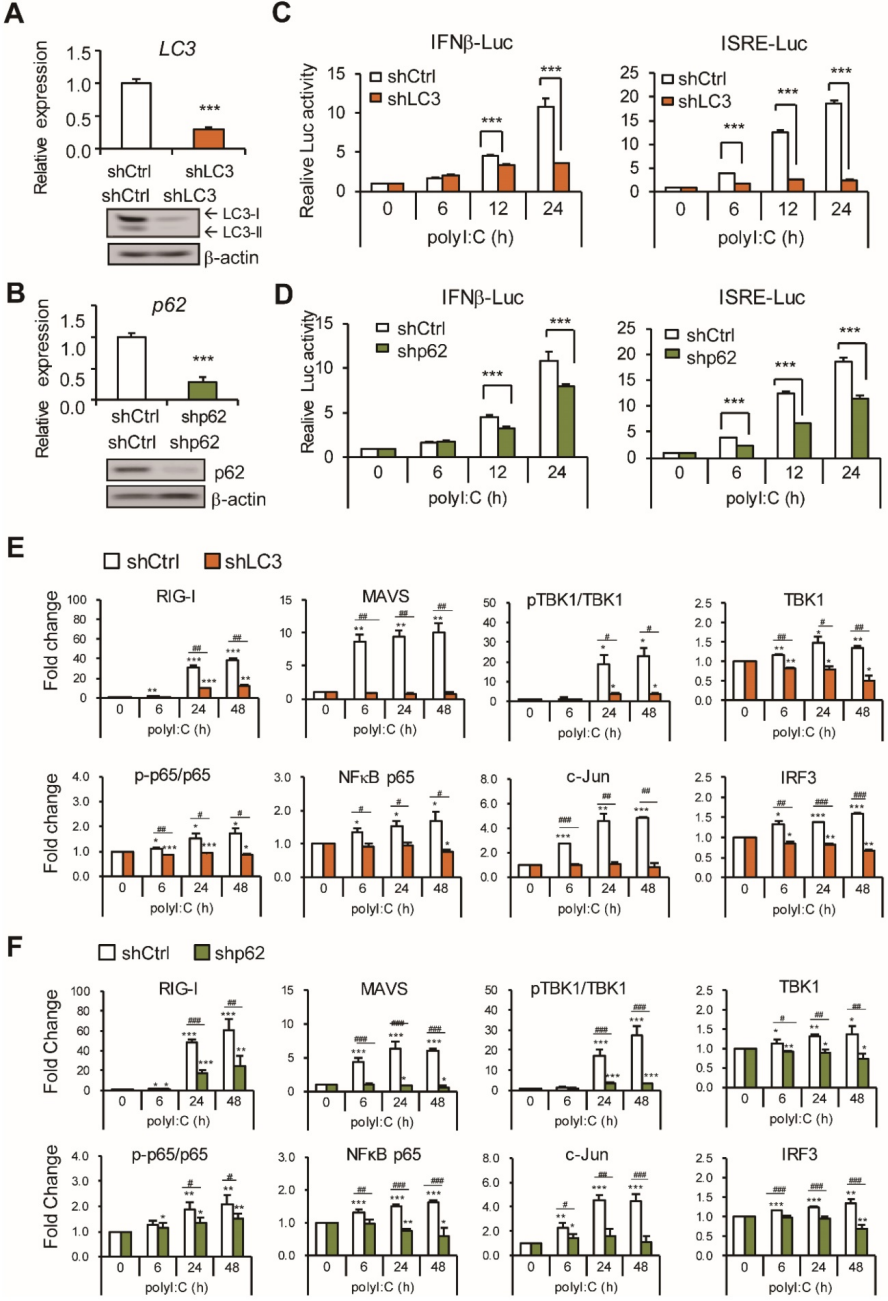
** Knockdown of LC3 and p62 downregulate RLR activity. (A-B)** The knockdown efficiency of LC3 and p62 was measured using RT-qPCR (upper panel). Relative mRNA level was calculated with reference to *Gapdh*. Data are mean±SD from three independent tests; ***, P < 0.001 vs shCtrl. The knockdown LC3 and p62 protein expression was confirmed by immunoblotting assay (lower panel). β-actin was a loading control. **(C-D)** IFNβ and ISRE-luciferase reporter assays were conducted in LC3-, p62-knockdown or shCtrl cells after polyI:C (2.5 μg) stimulation, the dual-luciferase assay was measured at the indicated time points. Data are presented as mean±SD from three independent tests; ***, P < 0.001 vs shCtrl. **(E-F)** Immunoblotting assay of RLR signaling in Ctrl or LC3, p62 knockdown A549 cells with polyI:C stimulation. RLR protein levels from immunoblotting were quantified with the loading control β-actin. Data are mean±SD from three independent tests; * P, < 0.05, **, P < 0.01, ***, P < 0.005 vs without polyI:C stimulation. ^#^, P < 0.05,^ ##^, P < 0.01, ^###^, P < 0.005 comparing between shCtrl and shLC3 with polyI:C stimulation.

To further validate the role of LC3 and p62 in the type I IFN induction pathway, LC3 and p62 mRNA and proteins were knocked down by retrovirus-shRNA transduction in A549 cells (Figure 3A-B). IFNβ and ISRE reporter assays showed significant reduction of polyI:C-induced luciferase activity in LC3- and p62-knockdown cells (Figure 3C-D). RLR signaling in LC3- and p62 knockdown A549 cells was evaluated with polyI:C stimulation (Figure 3E-F and Figure S2A-B). As compared with unstimulated cells, LC3 and p62 knockdown downregulated polyI:C induced protein level of RIG-I, MAVS, TBK1, IRF3, NFκB p65 and c-Jun; also the ratio of the phospho-TBK1 to total TBK1 and phospho-NFκB p65 to total NFκB p65.

**Figure 4:**
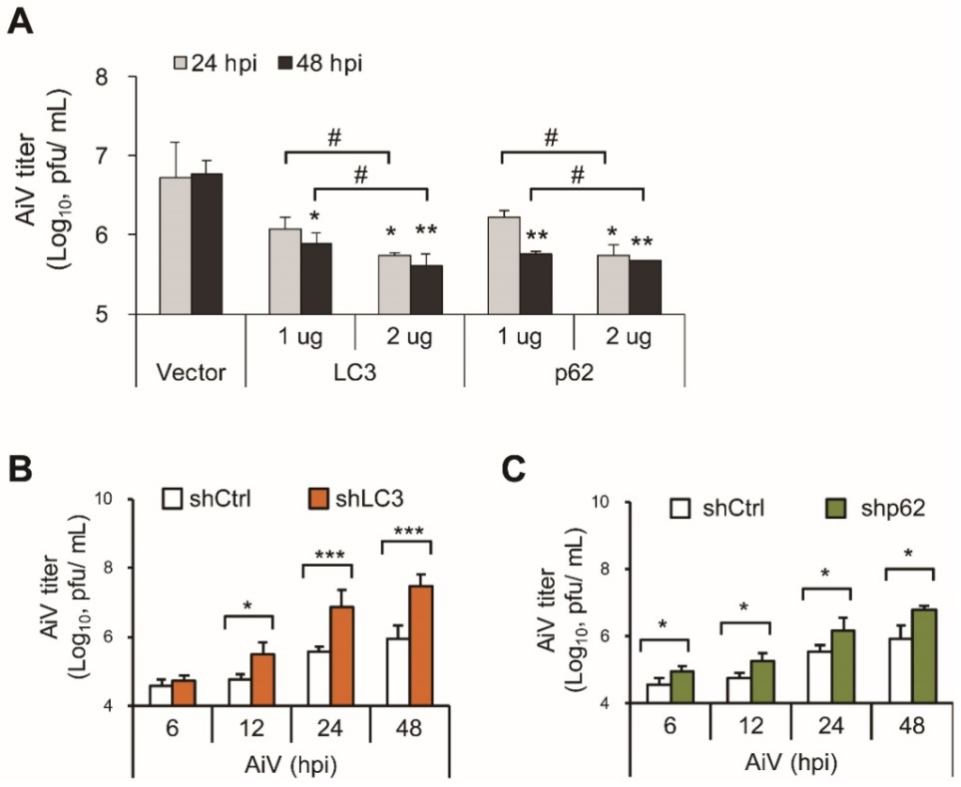
** LC3 and p62 attenuate AiV replication. (A)** LC3 and p62 or control vectors were transiently transfected into A549 cells for 24 h, which were then infected by AiV (MOI 5) for 24 and 48 h. The culture supernatant was harvested for plaque assay of virus titration. *, P < 0.05, **, P < 0.01, vs control vector. ^#^, P < 0.05 comparing 24 and 48 h post infection. **(B and C)** Plaque assay of the supernatants from control, LC3 and p62 knockdown cells with AiV (MOI 5) infection. Dara are mean±SD from three independent tests; *, P < 0.05, **, P < 0.01, ***, P < 0.005.

The present findings suggested that LC3 and p62 contribute to the RLR pathway to restrict viral infection. Thus, LC3 and p62 ectopic overexpressed or knockdown cells were infected with AiV to evaluate the antiviral effect of LC3 and p62. Virus titration showed that overexpression of LC3 and p62 significantly suppressed AiV production at 48 h post-infection as compared with the vector control (Figure 4A). On the contrary, AiV replication was time-dependently increased in LC3- and p62-knockdown cells (Figure 4B-C). These results indicated the involvement of LC3 and p62 in the RLR antiviral machinery. AiV may subvert LC3- and p62-associated antiviral defense machinery for its replication.

### AiV 3C modulates the RIG-I-IRF3 pathway

Results obtained showed that AiV infection induced a low level of RIG-I/IRF3 and LC3/p62 signaling activation, resulting in a low level of type I IFN production (Figure 1), and implying downregulation of RLR signaling by AiV for its replication. Indeed, we found that AiV attenuated the polyI:C-induced IFNβ-luciferase activity and RIG-I, MDA5, IRF7 and phosphor-IRF3 expression (Figure S3A-B). Moreover, RIG-I activated IFNβ luciferase activity was reduced by AiV infection by downregulating RIG-I expression (Figure S3C-D), evidencing the ability of AiV to weaken the antiviral defense in cells 35.

**Figure 5:**
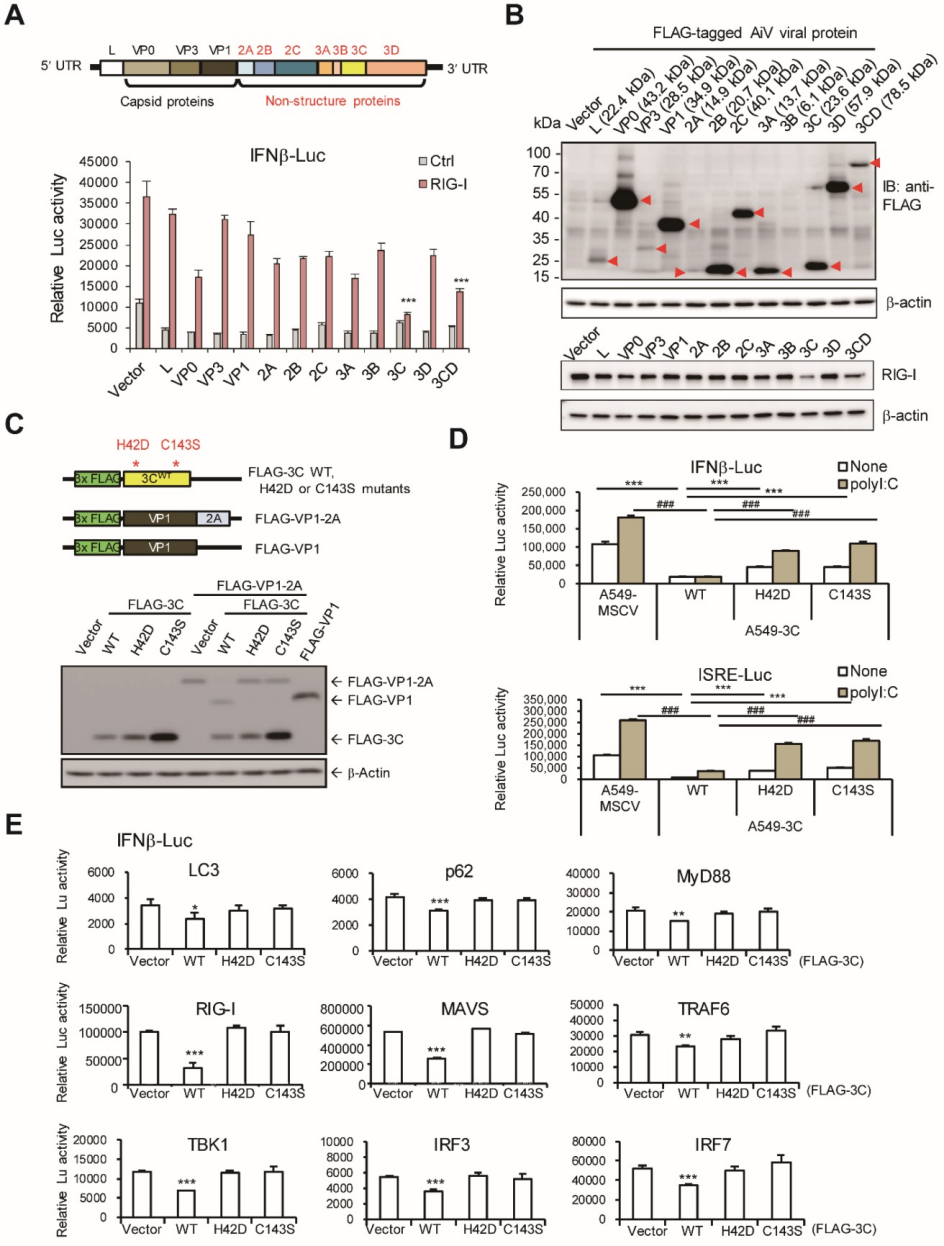
** AiV 3C protease inhibits RLR activity. (A)** Upper panel shows the AiV genome composition. Lower panel, A549 cells (2 × 10^5^) were cotransfected with IFNβ luciferase reporter plasmid and pRL-TK along with the RIG-I and viral protein expression vectors for 24 h, then, dual-luciferase assay was performed. **(B)** Cell lysates from panel (A) were subjected to immunoblotting assay, the viral protein and RIG-I expression were detected by antibodies against anti-FLAG and RIG-I. **(C)** Schematic diagram of FLAG-tagged AiV 3C protease WT, 3C protease dead mutants (H42D and C143S), VP1-2A fusion and VP1 vectors (upper panel). The Protease activity of 3C WT and mutants was determined by cotransfection with FLAG-VP1-2A; FALG-VP1 was the cleavage control (lower panel). **(D)** IFNβ and ISRE luciferase reporter assay of A549 cells with ectopic expression of 3C WT and mutants. Cells were stimulated with polyI:C (2.5 μg/ml) for 24 h then underwent dual luciferase assay. Data are mean±SD from three independent tests; ***, P< 0.001, vs None-treatment group of A549-3C WT, ^###^, P < 0.001, vs polyI:C stimulation group of A549-3C WT. **(E)** IFNβ luciferase reporter assay was conducted by co-transfection of indicated RLR signal protein vectors and 3C WT or protease mutants in A549 cells. After 48 h of transfection, dual luciferase activity was measured. Data are mean±SD from three independent tests; * P, < 0.05, **, P < 0.01, ***, P < 0.005.

The AiV polypeptide consists of protein L, VP0, VP3, VP1, 2A, 2B, 2C, 3A, 3B, 3C and 3D or 3CD (Figure 5A upper panel). To identify which viral protein obstructs RLR, AiV viral protein was cloned and fused with a FLAG tag for IFN-β reporter assay. Results showed significant reduction of RIG-I-induced reporter activity by AiV 3C and 3CD proteins (Figure 5A lower panel). In view of low inhibition activity exhibited by AiV 3D, AiV 3C protease may play a critical role to suppress type I IFN production. Immunoblotting confirmed the viral protein expression with proper molecular size (Figure 5B upper panels). In addition, RIG-I protein level was downregulated by 3C (Figure 5B lower panels). AiV 3C had a dose- dependent effect on the IFN-β reporter inhibition and RIG-I protein degradation (Figure S3E).

### AiV 3C protease activity required in modulating type I IFN response

AiV 3C protease dead mutants were constructed to evaluate its protease activity in regulating antiviral activity (Figure 5C, upper panel). The protease activity of AiV 3C wild type (WT) and mutants was confirmed by cotransfection with VP1-2A fusion protein in cells. The VP1 protein dissociated from the VP1-2A fusion protein was detected 3C WT but not 3C protease dead mutants H42D and C143S. FLAG-VP1 served as control (Figure 5C, lower panel). A549 cells with AiV 3C WT and mutants by retrovirus transduction were subjected to reporter assay. Results showed 3C-suppressed polyI:C-mediated IFN-β and ISRE luciferase activity alleviated by the 3C protease dead mutants (Figure 5D). In addition, IFN-β luciferase signaling activated by LC3, p62, RIG-I, MyD88, MAVS, TRAF6, TBK1, IRF3 and IRF7 was downregulated by 3C WT protease but not 3C protease dead mutants (Figure 5E).

### AiV 3C protease activity downregulates signaling protein expression

**Figure 6:**
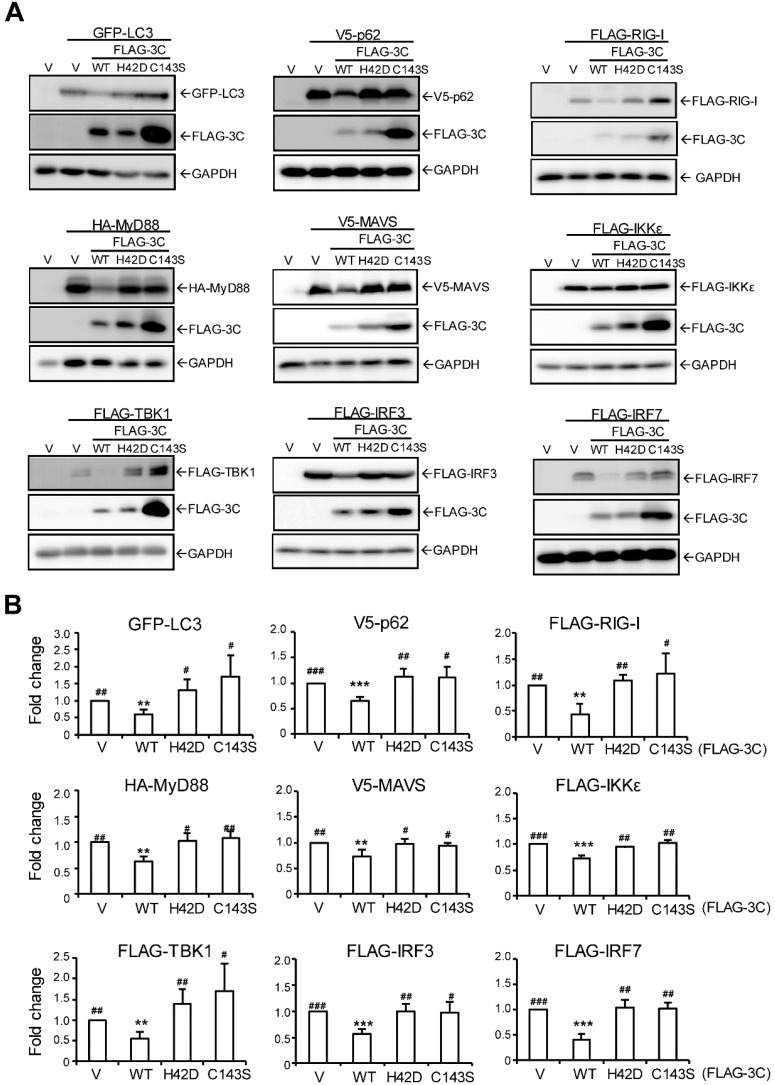
** AiV 3C protease downregulates RLR signal protein expression. (A)** AiV 3C WT or protease dead mutant (H42D and C143S) plasmids (2 μg) were cotransfected with the indicated RLR signal protein expression vector (2 μg) in A549 cells for 24 h. Ectopic protein expression was analyzed by immunoblotting analysis with anti-GFP, anti-V5, anti-FLAG or anti-HA antibody. **(B)** RLR protein expression level from immunoblotting (A) was quantified with the loading control β-actin. Data are mean±SD from three independent tests; *, P< 0.05, **, P < 0.01, ***, P < 0.005 vs without polyI:C stimulation. ^#^, P < 0.05,^ ##^, P < 0.01, ^###^, P < 0.005 comparing shCtrl and shLC3 with polyI:C stimulation.

To understand whether 3C protease downregulates LC3 and p62 and other signal activator proteins to control IFN production activity, cells were co-transfected with 3C WT or mutants with signal proteins. Immunoblots of LC3, p62, RIG-I, MyD88, MAVS, IKKε, TBK1, IRF3 and IRF7 showed that 3C WT but not 3C protease dead mutants downregulated each signal protein expression (Figure 6A). The fold change in protein level (vs. vector control) confirmed that AiV 3C protease is required to diminish RLR signaling (Figure 6B).

## Discussion

This study investigated the role of LC3- and p62-associated RLR against AiV. Results revealed that AiV infection induced a moderate level of host antiviral and inflammation response in comparison with polyI:C dsRNA stimulation. AiV-induced degradation of autophagosome-associated proteins LC3 and p62 was not observed in polyI:C-stimulated cells. PolyI:C or autophagosome blockers accumulated LC3 and p62 proteins, which activated RLR activity against AiV infection. This result was consistent with the LC3 and p62 overexpressed cells. In contrast, knockdown of LC3 and p62 downregulated the activity of polyI:C-mediated RLR signaling and resulted in a low level of type I IFN and ISRE reporter signaling, which caused enhanced AiV virion production. Furthermore, the present findings revealed the significant role of AiV 3C protease in dampening host antiviral machinery. Type I IFN production promoted by polyI:C, LC3, p62 or RLR signaling proteins was downregulated by 3C protease WT but not protease dead mutants.

Autophagy pathway and proteins play critical roles in adaptive and innate immunity against pathogens 17. Our data showed that autophagosome inhibitors (HCQ and BAF-A1) inhibited AiV infection as well as IFNβ and NFκB reporter activities. A possible mechanism might be associated with LC3 and p62 protein accumulation-mediated RLR activation (Figure 2). Our previous study also showed that autophagosome inhibitor HCQ activates type I IFN expression against dengue virus, which supports positive regulation of innate immunity by late-stage autophagy flux 32. However, Atg5 and Atg16L may not contribute to innate antiviral immunity. Activation of IFNβ or other tested reporters, and TBK1-IRF3 signaling were not detected in cells with Atg5 and Atg16 overexpression (Figure S1). These results are supported in part by the previous finding that Atg5-Atg12 conjugates regulated negatively type I IFN production pathway by direct binding of RIG-I and MAVS 22. Enterovirus 71 non-structural protein 2C was found to defeat host anti-viral factor APOBEC3G (A3G) suppression by the induction of A3G degradation through the autophagy-lysosome pathway 36. In our study, LC3- and p62-mediated antiviral innate immunity may be a non-canonical autophagy mechanism independent of an autophagosome-associated degradation pathway. LC3 activating type I IFN production was supported by a previous study in which LC3 interacted with IKKα to activate IRF7 for IFN synthesis 33. Another mechanism of LC3-restricted viral replication considers the degradative autophagy flux unnecessary, but may be related to LC3-guided IFN inducible immunity-related GTPases 37.

Here, we observed that overexpression of p62 or blocking p62 degradation positively regulated antiviral inflammatory response against AiV, and knockdown of p62 downregulated type I IFN expression (Figs. 2-4). These data are consistent with a previous finding the basal level of type I IFN reduced in p62-knockdown HeLa cells without additional stimulation 38. Mechanistic study suggested that p62 stabilizes the oligomerization of pattern-recognition receptor NOD2 to enhance NOD2-mediated signaling and cytokine production activity 39. Thus, p62 serves as an activator in the host defense innate immunity.

*Enterovirus* genus of the *Picornaviridae* family, such as enterovirus 71 or 68, poliovirus and coxsackievirus virus B3, have evolved diverse strategies to evade the IFN response for their replication in cells. Signal proteins, RIG-I, MDA5, MACS, TRIF, IRFs, IKKs, and NF-κB in the pathogenic recognition receptor (PRR)-mediated signaling pathway are targeted for cleavage or degradation by enterovirus viral protease 40. This study is the first to report that a *Kobuvirus* AiV, and its 3C protease use a similar strategy to downregulate PRR signaling. The current results suggest that the protease of picornaviruses is the critical viral factor in subverting host antiviral response. However, further exploration of the direct cleavage or indirect degradation effect of AiV 3C protease is required to evaluate whether or which ubiquitin-mediated degradation is involved in downregulating the host antiviral pathway.

The dual effects of autophagy which promoted the clearance of viral components and activated antiviral cytokine production during virus infection was emphasized 41. Coxsackievirus B3 protein modulates NFκB activity by cleavage of p62 42. In this study, LC3 and p62 protein degradation mediated by AiV- and its 3C protease-mediated play a critical role in blocking signaling activity of the RIG-I-IRF3 axis. The present findings reveal viral factors interfering in the cross-talk between autophagy and type I IFN responses.

In conclusion, AiV can downregulate the LC3- and p62 mediated RLR antiviral signaling pathway by its 3C protease activity. In addition to suppressing 3C protease activity, maintaining the cellular level of LC3 and p62 may promote host antiviral activity against AiV infection.

## Supplementary Material

Supplementary figures and tables.

## Abbreviations

3-MA: 3-Methyladenine
AiV: Aichi virus
AP-1: activator protein 1
ATG: autophagy-related protein
BAF-A1: bafilomycin A1
CARD: caspase recruitment domain
CQ: chloroquine
DMEM: dulbecco's Modified Eagle Medium
dsRNA: double-stranded RNA
HEK293T: human embryonic kidney 293T
HCQ: hydroxychloroquine
IFN: interferon
IKKε: inhibitor of κB kinase ε
IRF: interferon regulatory factor
ISG15: interferon-stimulated gene 15
ISRE: interferon-sensitive response element
LC3: microtubule-associated protein light chain
LGP2: laboratory of genetics and physiology gene 2
MAVS: mitochondria antiviral signaling protein
MDA5: melanoma differentiation-associated protein 5
MEF: embryonic fibroblasts
MOI: multiplicity of infection
MSCV: murine Stem Cell Virus
mTOR: mammalian target of rapamycin
MyD88: myeloid differentiation primary response 88
pDC: plasmacytoid dendritic cells
PI3K: phosphoinositide 3-kinase
RIG-I: retinoic acid-inducible gene-I
RLR: retinoic acid-inducible gene-I like receptor
RSV: respiratory syncytial virus
shRNA: short hairpin RNA
SQSTM1: sequestosome-1
TBK1: TANK Binding Kinase 1
TLR: toll-like receptor
TRAF6: tumor necrosis factor receptor-associated factor 6
TRIM: tripartite motif containing proteins
ULK1: unc-51 like autophagy activating kinase 1
VP: viral protein
VSV: vesicular stomatitis virus
