# Supplementary material for: Aichi virus 3C protease modulates LC3- and SQSTM1/p62-involved antiviral response
Source: Theranostics. 2020 Jul 14;10(20):9200–13. doi: 10.7150/thno.47077 (PMC7415801; doi:10.7150/thno.47077)
Supplement: Supplementary file 1 — Supplementary figures and tables. [file thnov10p9200s1.pdf]

Supplementary information

Aichi virus 3C protease modulates LC3- and SQSTM1/p62-involved antiviral response

Ming-Hsiang Kung, You-Sheng Lin, Tsung-Hsien Chang

Table S1. Primer sequences for AiV viral gene cloning.

Target	Primer sequences (5' to 3')		Cloning site
AiV-L	F	CCGCGAATTCA **ATG** GCTGCAACACGGGTTTCA	EcoR I
	R	CCCGGGATCC TCA TTGCCGTTGGAGGTTGGT	BamH I
AiV-VP0	F	CCGCGAATTCA **ATG** GGCAACTCGGTCACCAAC	EcoR I
	R	CCCGGGATCC TCA CTGTGGGGCGAGGTAG	BamH I
AiV- VP3	F	CGATAGATCTG **ATG** CACTGGAAGACTCGCACC	Bgl II
	R	CCCGGGATCC TCA CTGGGAAGTGAGGGCAG	BamH I
AiV- VP1	F	CCGCGAATTCA **ATG** ACCCTCACCGAAGACCTC	EcoR I
	R	CCCGGGATCC TCA GTAGGTGGGGCGCTG	BamH I
AiV- 2A	F	CCGCGAATTCA **ATG** GTCCACTGGGCCATCC	EcoR I
	R	ATCCTCTAGA TCA CTGTCGCCTGATGCCTGG	Xba I
AiV- 2B	F	CGATAGATCTG **ATG** GGCCTCCTCACCCTCT	Bgl II
	R	CCCGGGATCC TCA TTGAGGTTCAAGGGTTGCC	BamH I
AiV- 2C	F	CCGCGAATTCA **ATG** GGGCTCAAAGACTTCAACG	EcoR I
	R	CCCGGGATCC TCA CTGGCGTTTGATGAGGGA	BamH I
AiV- 3A	F	CCGCGAATTCA **ATG** GGTAACCGGGTCGTCG	EcoR I
	R	CCCGGGATCC TCA TTGGGGTTCCCGTGGC	BamH I
AiV- 3B	F	CCGCGAATTCA **ATG** GCTGCCTACTCTGCTATC	EcoR I
	R	CCCGGGATCC TCA TTGGCGCTGGATGTGGC	BamH I
AiV- 3C	F	CCGCGAATTCA **ATG** GGAATCTCCCCCGCTG	EcoR I
	R	CCCGGGATCC TCA TTGCTGGGTGGTGGCAAAT	BamH I
AiV- 3D	F	CCGCGAATTCA **ATG** TCTCTCATTGTCCCCACTG	EcoR I
	R	CCCGGGATCC TCA GGCAGCCAGCAGATTGAG	BamH I
AiV-3C H42D		TACCTTCTGGTCCCCACCGACCTCCGTGAACCCCA	Site-directed mutagenesis
AiV-3C C143S		CGACCTTCGAAGGTCTGTCCGGATCCCCGCTTGT	Site-directed mutagenesis

F: forward primer; R: reverse primer

Bold letter: Insert other nucleotides for transcription start site

Below line: Insert restriction enzyme sequences for gene cloning

Table S2. Primer sequences for qPCR.

Viral gene	Primer sequences (5' to 3')	
Human IFNα1	F	CCTCGCCCTTTGCTTTACTG
	R	GCCCAGAGAGCAGCTTGACT
Human IFNβ	F	TGA GCA GTC TGC ACC TGA AA
	R	GCT TGA AGC AAT TGT CCC GT
Human RIG-I	F	GCA GAG GCC GGC ATG AC
	R	TGT AGG TAG GGT CCA GGG TCT TC
Human MDA5	F	TGC TTC TCT AAG TGG GCA GC
	R	TTT TCA CCC TGG CCC TGA AG
Human TBK1	F	GGA GAC CCG GCT GGT ATA A
	R	TGA ACA TCC ACT GGA CGA AGG
Human IRF3	F	GAC CTT CCA TCG TAG GCC G
	R	AAT CCT CCT GCT GTG CAT CC
Human IRF7	F	AGC TGT GCT GGC GAG AAG
	R	TGG AGT CCA GCA TGT GTG TG
Human IKKε	F	AAG AGC CGG GAT CAG GTA CA
	R	CAT CTT GTC CAA ACA GCA CTG AA
Human ISG15	F	GGT GGA CAA ATG CGA CGA A
	R	ATG CTG GTG GAG GCC CTT A
Human IFIT3	F	GCT GAA GGA GAG CAG TTT GTT GA
	R	AGG ACA TCT GTT TGG CAA GGA
Human Viperin	F	CAA GGA AGA ATG TGA GCA AGA GTA GA
	R	TGA TAT GGT GAC ATG GCT TCA CT
Human MyD88	F	GAG CTG GCG GGC ATC AC
	R	TCG AAA CGC TCA GGC ATA TG
Human Trim5α	F	GCC TGG AAC TCC TGA CAC AAC
	R	CAT GGA CTT CTT GTG GTT TGC A
Human Trim25	F	CGA GGT GGA ACT GAA CCA CA
	R	GTG GAT TTG TGT GTG GAC GC
Human LC3	F	GGC GCT TAC AGC TCA ATG C
	R	ACC ATG CTG TGT CCG TTC AC
Human p62	F	CCA TGT CCT ACG TGA AGG ATG A
	R	CCG CCG GCA CTC TTT TT
Human TNFα	F	TGC TCC TCA CCC ACA CCA T
	R	GGA GGT TGA CCT TGG TCT GGT A
Human IL-6	F	GCT GCA GGC ACA GAA CCA
	R	GCT GCG CAG AAT GAG ATG AG
Human IL-8	F	CTG GCC GTG GCT CTC TTG
	R	CTT GGC AAA ACT GCA CCT TCA
Human CXCL10	F	CCT GCA AGC CAA TTT TGT CCA
	F	TGC ATC GAT TTT GCT CCC CT
Human GAPDH	F	CAA CTG GTC GTG GAC AAC CAT
	R	GCA CGG ACA CTC ACA ATG TTC

F: forward primer; R: reverse primer

Figure S1

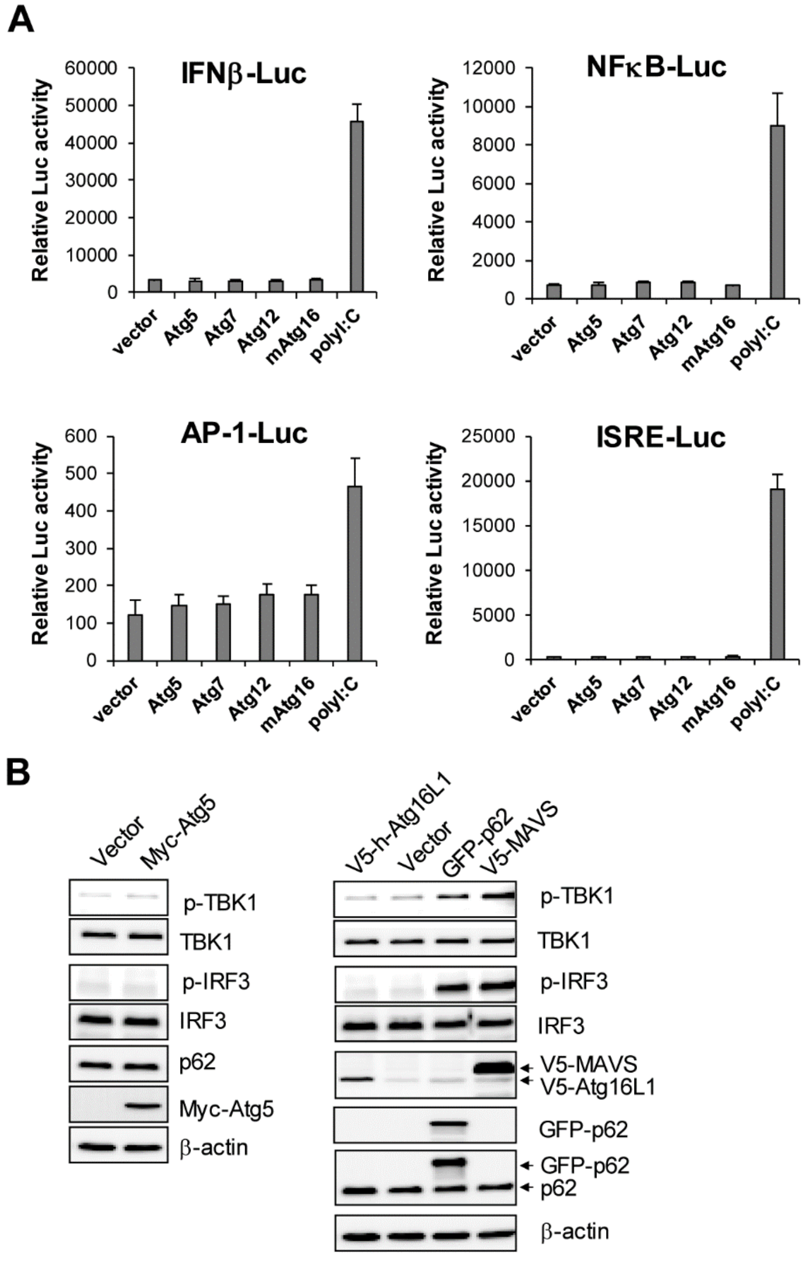

Figure S1. **Evaluation of regulation effect of Atg conjugation system in RLR pathway. (A)** Luciferase reporter assay of IFNβ, NFκB, AP-1 and ISRE in A549 cells (1×10^5) with overexpression of Atg5, Atg7, Atg12 and mAtg16L1 expression vectors or polyI:C stimulation for 24 h. **(B)** Immunoblotting assay of RLR signaling, LC3 and p62 protein levels in A549 cells with Atg5, or mAtg16L1. GFP-p62 and V5-MAVS were the positive control.

Figure S2

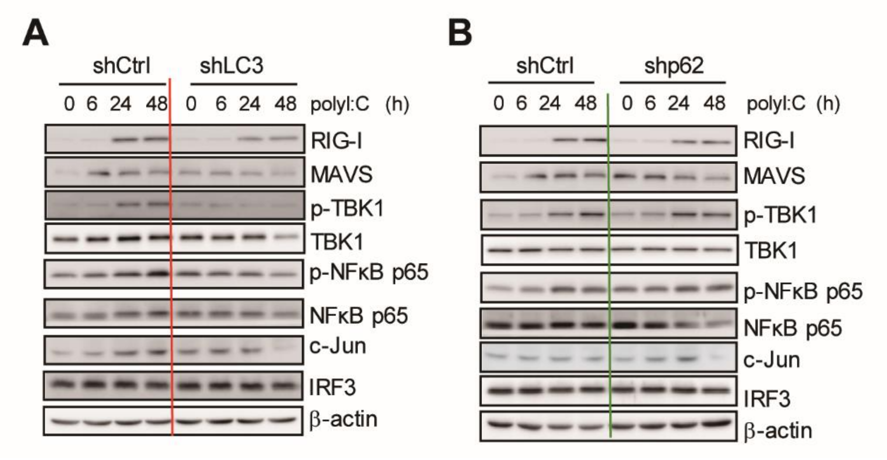

Figure S2. Analysis of RLR signaling in LC3- and p62-knockdown cells. (A and B) Immunoblotting assay of RLR signal proteins in shCtrl, shLC3 or shp62 A549 cells with polyI:C stimulation.

Figure S3

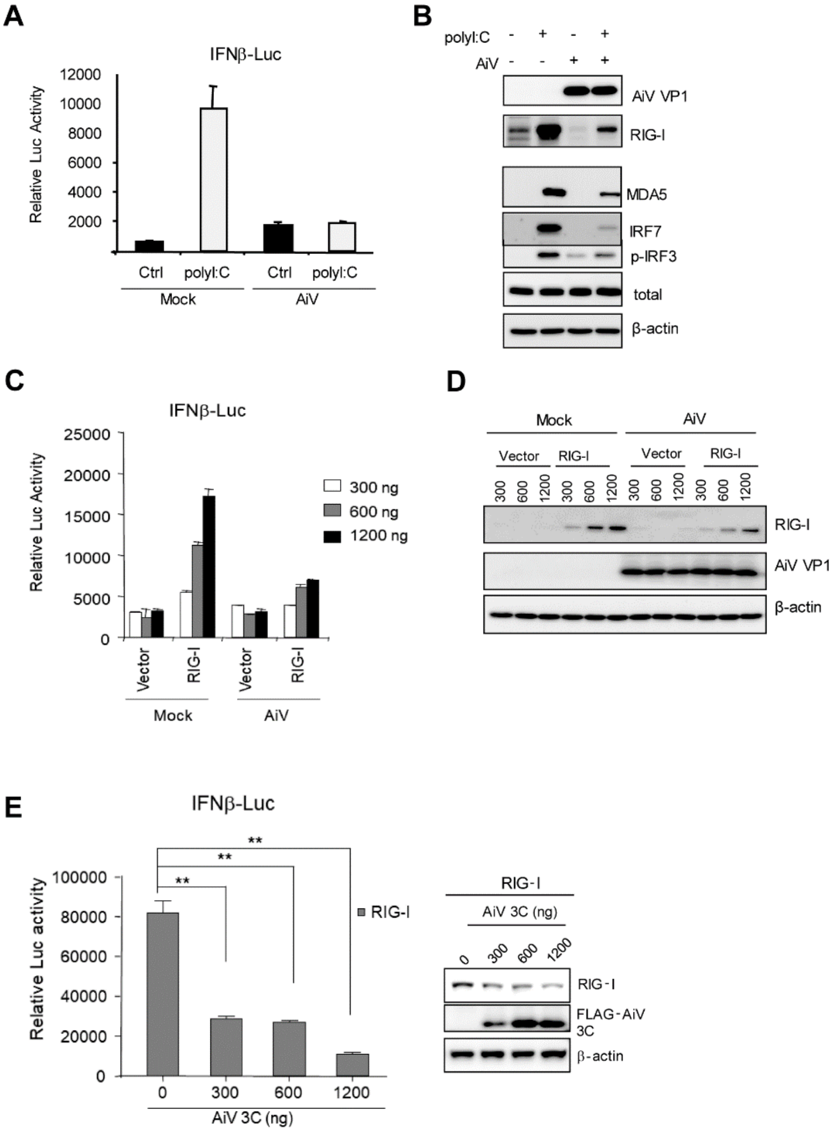

Figure S3. AiV attenuates polyI:C-promoted RLR response. (A) A549 cells were cotransfected with IFNβ-Luc reporter and pRL-TK for 24 h, and then infected by AiV (MOI = 1). Cells were then stimulated with polyI:C for 24 h and dual luciferase assay was performed. **(B)** Mock- or AiV-infected A549 cells were stimulated with polyI:C, cell lysates were subjected to immunoblotting with the indicated antibody. **(C)** IFNβ-Luc reporter, pRL-TK and RIG-I expression vector (300, 600, 1200 ng) were transfected into A549 cells for 24 h, which were then infected by AiV (MOI 1). Dual luciferase assay was performed at 24 h after infection. Data are mean±SD from three independent tests. RIG-I expression and AiV VP1 expression are shown in panel **(D)**. **(E)** A549 cells were co-transfected with RIG-I expression vector (1200 ng) and AiV 3C vector (300, 600, 1200 ng). Post-transfection 24 h, cell extracts underwent immunoblotting analysis.
